# Metropolitan Hygiene in the Past
*Stow's Survey of London. By Strype. London: 1720.Londinium Redivivum. By James Peller Malcolm. London: 1803.The History and Art of Warming and Ventilating Rooms and Buildings. By Walter Bernan, Civil Engineer. London: 1845.Archæologia, vol. xx.Natural and Political Observations made upon the Bills of Mortality. By Capt. John Graunt, F.R.S. (Sir William Petty.) London: 1676.Orders thought meete by Her Majestie, and her Priuie Counsell, to be executed throughout the Counties of this Realme, in such Townes, Villages, and other places, as are, or may be hereafter infected with the Plague, for the stay of further increase of the same. London: 1592.Certain Necessary Directions, as well for the Cure of the Plague, as for preventing the Infection. London: 1636.A Collection of very valuable and scarce Pieces relating to the last Plague, in the year 1665. London: 1721.


**Published:** 1858-01

**Authors:** 


### METROPOLITAN HYGIENE IN THE PAST.*

In an era when the public intelligence is awake to the importance of sanitary measures; when the truth that the health of the community has been entrusted in a great measure to the keeping of the community seems to be obtaining a hold on the minds of men; when sanitary commissions are appointed, and sanitary officers are salaried; when voluminous blue books are printed by Government, and modest reports by vestry boards; when noble lords utter sanitary addresses, and ignoble com-

---

* Stow's Survey of London. By STRYPE. London: 1720.

Londinium Redivivum. By JAMES PELLER MALCOLM. London: 1803.

The History and Art of Warming and Ventilating Rooms and Buildings. By WALTER BERNAN, Civil Engineer. London: 1845.

Archæologia, vol. xx.

Natural and Political Observations made upon the Bills of Mortality. By Capt. JOHN GRAUNT, F.R.S. (Sir William Petty.) London: 1676.

Orders thought meete by Her Majestie, and her Priuie Counsell, to be executed throughout the Counties of this Realme, in such Townes, Villages, and other places, as are, or may be hereafter infected with the Plague, for the stay of further increase of the same. London: 1592.

Certain Necessary Directions, as well for the Cure of the Plague, as for preventing the Infection. London: 1636.

A Collection of very valuable and scarce Pieces relating to the last Plague, in the year 1665. London: 1721.

moners disburse sanitary rates, a glance at the hygienic condition of the great towns of our empire in bygone times may present some material for comparison and reflection, and will at least afford us no insignificant ground for congratulation.

In the present article, we do not pretend to give anything like a connected history; such a work would extend far beyond the limits of a review. We only hope to lay before our readers some few historical facts, frequently disconnected with each other, some of which may be found suggestive to the England that is, some merely illustrative of the England that was.

In turning over our annals, one fact in relation to the subject forces itself upon our attention; it is that the eras marked by sanitary reforms, have been the eras of great pestilences. We owe our present movement to the cholera, as our ancestors owed similar ones to the plague and other epidemics. For centuries the mortality in our population was far higher than at present, and the increase was proportionally far less. Much is to be allowed for the effects of civil wars, of vindictive and oppressive legislation, and of popular excesses. But, making ample deductions for these, we may safely assert that human life was sacrificed wholesale to ignorance and inertness; and it was not until some great visitation swept off the people in masses that their rulers were aroused to a temporary exertion, and that they themselves were terrified into a temporary reform.

Another fact that strikes the inquirer, is the frequent similarity existing between the hygienic measures pursued in former ages, and those at present so widely promulgated. It is no new discovery that the presence of filth and offal is prejudicial to health, that cattle markets and slaughter-houses should be extra-urban, that the water supply should be pure, that sewerage and drainage are indispensable, that the resting places of the dead should be removed from the living. These things were acknowledged to be truths by the better instructed in what we are accustomed to consider comparatively dark ages; but, as in our own times, they were not acted on until survivors were aroused from their apathy by some great devastation.

To illustrate what we have said. The fourteenth century was marked by three fearful epidemics, known to historians as the first, second, and third mortalities. The first was the celebrated black death, the most awful pestilence the world has ever known. After the second, which occurred in the year 1361, and which was either of the same nature as the former, or, as is more generally believed was an irruption of the ordinary bubo plague, Edward III, in Parliament, issued a proclamation forbidding the slaughter of cattle in the city of London, on account of the pollution of the streets and sewers thence arising, which was supposed to foster the pestilence. It was further ordered, that the killing of meat for the city should be confined to the precincts of Stratford and Knightsbridge.

Three hundred years afterwards, in 1636, the plague again swept over our capital, carrying off 10,460 people. The reigning monarch, Charles I, applied to the College of Physicians for advice and instructions. In the recommendations of the College, drawn up by royal authority, we read: "It were also to bee wished that the Slaughter-houses were vtterly put from out the liberties of the City, being in themselues very offensiue; and that funnells in Church-vaults be considered of, and the depth of graues." In the great plague of 1665, the same advice was repeated.

Two centuries elapse, marked by revolutions, political, social, and intellectual. The plague has long become extinct in our land. Another wide spreading disease attacks the population; and its onslaught is repeated and repeated again with alarming rapidity. The powers that be have again recourse to the men of medicine; and, with provoking sameness, in the year 1854, we find from Dr. Sutherland's report the slaughter-houses still existing in crowded neighbourhoods, and referred to, rightly or wrongly, as causes of local outbreaks of cholera. Verily, the English are a people not given to change.

We would not be supposed from these remarks to undervalue the great sanitary improvements of modern times, or to attribute to our ancestors a greater perfection in the art of living than that to which they had arrived. All that we intend to show is, that many of the same imperfections in our system have been allowed to descend unaltered to our own times, and that a want of enlightenment in former generations cannot always be pleaded as apology.

In order that we may give our readers an idea of what has been accomplished, we have endeavoured to group together a few facts connected with sanitary matters as they have existed since the era of the Norman Conquest; and we shall principally, though not exclusively, confine our descriptions to the heart of the empire, the great metropolis.

The oppression and cruelty which accompanied and succeeded the Conquest, found their results in the hygienic condition of the people. It is admitted by all, that the towns suffered directly less under the rule of the conquerors than the country districts; but, in consequence of the ravages of sword and fire, the excessive taxations, and the cruel forest laws, agriculture was at an end: whole districts, previously smiling in peace and overflowing with plenty, were depopulated, and the whole nation again and again experienced famine and its accompanying pestilence. What the resulting disease was, we have no certain knowledge. Sometimes it appears to have been dysenteric flux, sometimes fever, probably similar to that which occurred in Ireland after the failure of the potato crops. Space will not allow us to quote authorities; we would only refer the reader to the touching accounts given in the *Saxon Chronicle*, and scattered through the monkish historians.

But the Normans were a great people. Foremost in arms and first in council of all the races sprung from the teeming North, civilisation found in them her aptest scholars, and the arts their most enthusiastic votaries. Their refinement recoiled from the coarse gluttony and the boisterous drunkenness of the Saxon burgher and yeoman, whom they likened to the swine, which formed a large part of the wealth of the country and of the food of the town. The dominant race, however, were comparatively few in number; and it was long before hatred and poverty permitted the conquered to adopt in any degree the manners and modes of life of their conquerors. Still, we should err, did we not attribute to Norman influence many of those improvements which must eventually have affected the health and well being of the population.

In the ages immediately succeeding the Conquest, London was surrounded by a wall, which skirted along the river on the south side from the Tower to the Fleet, and on the north took the form of a crescent, with the exception of an indentation between the portals of Cripplegate and Aldersgate. Surrounding this wall was a ditch, continuous on the western side with the Fleet Ditch, or, as it was then called, the river of Wells, from the number of wells (which still give their names to the surrounding districts) whose overflowing waters augmented its stream. This ditch, which was originally 200 feet broad, formed the principal part of the existing sewerage of the city. It was commenced in the year 1211, and finished in the year 1213. Measures were constantly required from time to time, to keep this ditch free from accumulations of filth and offal, and also to prevent encroachments on it by the gardens and houses of the citizens. As late as the time of Elizabeth, we find various sums expended by the citizens for this purpose: but the earlier proceedings were evidently taken rather with the view of maintaining the defensive character of the fosse, and the navigation and wharfage of the Fleet river, which, in a petition of the fourteenth century, is said to have been wide and deep enough to carry ten or twelve ships as high as Fleet bridge, than from attention to its sanitary influence. Still, we find early proof of the recognition of the injurious effect on health produced by this vast open drain. In 1290, the Carmelite Friars, the Friars Preachers, and the Bishop of Salisbury, whose house then stood in Salisbury Court, complained to the King and Parliament of the putrid exhalations arising from the Fleet river, which were said to have occasioned the death of several of the brethren, and to be powerful enough to overcome the odour of the incense used in their services. One of the principal sources of its impurity at that time, was the great number of tanners' yards which existed in the neighbourhood.

Through all the vicissitudes which left their impress on old London, the enormous Fleet Ditch remained open to contaminate the air by its noxious exhalations; and it was not until the year 1736 that the great arch to carry off its waters was built, since which time its ancient course has marked one of the principal metropolitan sewers. We probably shall not do wrong, if we ascribe to its influence the high rate of mortality which obtained in the parishes on its banks during some of the great plague epidemics. Of this we subjoin a short table as a specimen.

| | In Plague Year 1593. | | In Plague Year 1625. | |
|---|---|---|---|---|
| | Died. | Of Plague. | Died. | Of Plague. |
| St. Andrew's Wardrobe, and St. Ann's, Blackfriars | 347 | 144 | 709 | 406 |
| St. Andrew's, Holborn | 1561 | 936 | 2190 | 1636 |
| St. Bride's | 897 | 607 | 1481 | 1031 |
| St. Sepulchre's | 3440 | 2502 | 3425 | 2420 |
| Total deaths from plague | | 4189 | | 5493 |

The total number of deaths from plague in London, in 1593, was 11,503, so that the district above named furnished more than one-third of the whole mortality; in 1625, when the deaths from plague amounted to 35,417, upwards of a seventh.

We are not to suppose that this great nuisance existed without frequently attracting public attention. Large sums of money were uselessly expended in attempting its purification. It furnished a frequent theme to the wits of a bygone age, and is immortalised in perhaps the most polished satire in the English language.

> "To where Fleet ditch with disemboguing streams,
> Rolls the large tribute of dead dogs to Thames,
> The king of dykes! than whom no sluice of mud
> With deeper sable blots the silver flood.
> Here strip, my children! here at once leap in,
> And prove who best can dash thro' thick and thin."
>
> DUNCIAD, b. II.

Our space will not permit us to give a succinct history of the sewerage of London. As population increased and civilisation progressed, the adoption of mere open water-courses was relinquished, and a better system was gradually introduced. After the great pestilence of 1603-4, we read in Strype's edition of Stow, that "Sir Leonard Halliday, maior anno 1606, laboured much for a river to be brought on the north of the city into it, for the cleansing the sewers and ditches; for the better keeping London wholesome, sweet, and clean. Sir John Walls, the next maior, seconded this good endeavour of Halliday. And one Nicholas Leate, a worthy and grave citizen, was very painful and industrious in the furtherance of this work, and the like therunto. And the city had in this year 1606, well cleansed their ditches and common sewers; and flood-gates were made in Holborn ditch and Fleet ditch." He afterwards goes on to inform us that "at this day, 1720, there be no ditches or boggs in the city except the said Fleet ditch, but instead thereof large common drains and sewers made to carry away the water from the postern gate between the two Tower Hills to Fleet bridge without Ludgate."

Before leaving the subject of sewerage, we may observe that the state of the Thames received special attention from our ancestors. In the time of Henry VIII, heavy penalties were imposed on those who contaminated the river by throwing soilage and offal into it. These substances were to be conveyed away in dung-boats to convenient places, to be appointed by the city authorities. Times, indeed, are changed. One of the great problems of the present age admitted then of an easy solution.

The water-supply of the citizens was first obtained from the natural brooks and wells which existed within their precincts. Of these there were several. Besides the river of Wells, which afterwards obtained the name of Turnmill brook, and its tributary the Old-Bourne, there was Wall brook, which entered the city near Moorgate, and, after various windings, fell into the Thames; then Langbourne, which rose in Fenchurch Street, and, running with a swift course west down Lombard Street, broke into many rills before its termination in the river. Together with these, there were the delicious springs of the suburbs. "There are," says an old writer, "on the north part of London, principal fountaines of water, sweet, wholesome, and cleere, streaming forth among the glistering pebble-stones; in this number Holy Well, Clerken-Well, and St. Clement's Well, are most famous, and frequented above the rest, when the schollers and the youth of the city take the air abroad in the summer evenings." As early, however, as the reign of Henry III, this primitive water-supply was found insufficient. In order "that the poor may drink, and the rich may dress their meat", the first water was conveyed from the town of Tyburn by pipes of lead into the city. A great leaden cistern, castellated with stone, to which the name of the great conduit was given, was erected in West Cheap for its reception; and other works of a similar nature followed. The water of the Thames was also used for domestic purposes; and the right of passage through the lanes which led to the river was held with tenacity by the citizens, in order that no interruption might be given to its carriage. To a Dutchman belongs the honour of first raising the Thames water and conveying it to the houses of the inhabitants by means of pipes; which he did by a mill, called in Stow "a most Artificial Forcier, standing near unto London Bridge." The name of the worthy Dutchman was Peter Morris, and the year of his achievement was 1582. He astonished the Lord Mayor and aldermen, when they came down to see his works, by throwing the water over St. Magnus' steeple. The water conveyed by this mill was long in use, and was deemed much clearer and finer than that supplied by the New River. This last named source of water-supply was brought to London in the year 1613, by Mr. Hugh Middleton, from the springs of Amwell and Chadwell; and it must have been of inestimable benefit to the metropolis, for its want was severely felt in the preceding reign. Queen Elizabeth had granted an Act of Parliament for the purpose of cutting such a river, but the project was dropped at her death.

We must now turn our attention from the water-supply to the habitations of our ancestors; introducing, as we go along, some few remarks on their modes of living.

The dwellings of the middle and lower orders in the Anglo-Norman times were constructed of wood, with plastered walls and thatched roofs. When inhabited by those whose circumstances were above poverty, they consisted of one large apartment with several smaller ones grouped around it: but the cottages of the poor contained only one room. The fire was made on a hearth in the centre, and the smoke found an exit through a central hole in the roof, or through the window when it existed. The houses in London were of this build, and about sixteen feet high. These apartments must have been as frequent a source of *lippitudo* as the smoky habitations which Lord Dufferin describes in Iceland. That such an effect was produced, we learn from Longlande, who wrote in the fourteenth century, and, although deploring the modern civilisation which had introduced chimneys, has left a testimony as to the discomfort of the primitive arrangement.

"Ac, when smoke and smorthre smyt in hus eyen,
Hit doth hym wors than hus wyf other where to slepe;
For thorw smoke and smorthre smerteth hus syghte,
Tyl he be blereyde othr blynde, and the borre in hus throte;
Kowgheth and corseth that crist ȝgive hym sorwe
That sholde brynge yn bettere wode, othr blowe til hit brente."

It is true that some chimneys were constructed in the stately castles of the great Norman barons, but several generations passed away before the peasantry and artizans departed from the style of their Saxon forefathers.

In Pierce the Plowman's Vision (from which the above is a quotation), it is a matter of complaint that the lord and lady abridged the ancient hospitality by leaving the smoky draughty hall, and retiring to a "privey parlowr," or a "*Chaumbre wyth a chymeney.*"

Many of the wretched cabins were destitute of windows. When these existed in the residences of both rich and poor, they were so small as to be almost useless, and lamps or candles were burned during the day. These eye-holes, as they were called by the Anglo-Saxons, were closed by linen or wooden lattice, or sometimes by thin plates of horn; glass was rare even in the castles of the conquerors. The ventilation of the habitations of both rich and poor must have been at a minimum, and to this probably is to be ascribed the love of perfumes which was so general. Fumigation was constantly and solely used as the means of purification and disinfection, and its substitution for fresh air was an error long adhered to both by the public and the profession. Witness the advice published by the College of Physicians, as late as the plague of 1665, where it is recommended that "Fumes of rosin, pitch, tar, turpentine, frankincense, myrrh, amber; the woods of juniper, cypress, cedars; the leaves of bays, rosemary; to which, especially to the less grateful scented, may be added somewhat of labdanum, storax, benzoin, lignum aloes; one or more of these as they are at hand, or may be procured, are to be put upon coals, and consumed with the least flame that may be, in rooms, houses, churches, or other places." But not one word is said about admitting any of the pure breath of heaven into the pestiferous apartments.

We regard the spread of the doctrine that fresh air is the best antagonist of disease, as the great sanitary improvement of modern times. Nearly every other of the acknowledged hygienic laws was recognised in greater or less degree, in past ages; but on this point our forefathers seemed completely ignorant. The belief in this truth has gradually been gaining ground since the time of Sydenham; but even now its vast importance is not fully appreciated, and it may be that its noblest triumphs are reserved to be witnessed by a future generation.

In the one-roomed huts of the lower orders, old and young slept round the central hearth. When a sleeping apartment existed, it served for the whole family, of whom several reposed in one bed. In the time of Chaucer, the packing system of the lodging houses in St. Giles's obtained in all respectable hostelries. That any traveller should have a room to himself was a mark of distinction; and down to a very late period, in farmhouses, inns, and servants' apartments, one great platform, sloping slightly from the head to the foot, served as a bedstead for a large number. A piece of furniture of this description, capable of accommodating fifty-two persons, existed till the middle of the last century at an ancient inn, the "Crown," at Ware, in Hertfordshire.*

To the crowding of students in the dormitories of the Universities, the great mortality occasioned by epidemic disease amongst them was doubtless rightly attributed. Thus, in 1448, when pestilence devastated Oxford, its great fatality was ascribed to "the lying of many scholars in one room or dormitory in almost every hall, which occasioned nasty air and smells, and consequently diseases."

The bed-rooms of the aristocracy possessed neither hearth nor flue. The tall narrow window was filled with oiled linen or glass, a part of which could be opened in the manner of a cottage casement, and the sweetness of the apartment was provided for by the burning of spices. The bedstead and furniture were luxurious for the age: a feather bed and pillow were laid on straw spread on the bed-laths, and "blanketts of fustyane" and "shetes of clothe of rayne," protected the sleeper. Over the bedstead a gilded cage hung aloft

> "With long peper fayre burning,
> And cloves that be swete smellyng,
> Frankensence and olibanum."

The state of the streets in great towns was such as would exist in the cities of the East in the present day, were it not for the sanitary labours of vultures and dogs. Reference has been already made to the proclamation of Edward III as to the removal of slaughter-houses to Stratford and Knightsbridge. It is evident that but little good effect resulted from so excellent an ordinance, for we find that in the reign of his successor, Richard II, a similar one was promulgated. The original proclamation sets forth that "By reason of killing of

---

* Twenty six butchers and their wives slept in it on the night of the coronation of William and Mary.

great beasts from *whose putrified blood running down the streets,* and the bowels cast into the Thames, the air in the city is very much corrupted and infected, whence abominable and most filthy stinks proceed, sicknesses and many other evils have happened to such as have abode in the said city, or have resorted to it; and greater dangers are feared to fall out for the time to come, etc." The reader will not wonder that the ancient name of the passage in which the Hall of the Butchers' Company stood was *Stinking Lane.* For evidence of the state of the streets in the middle ages we may turn again to the town of Oxford. "In 1300 the University complained to Edward I, that the great store of filth lying in the streets corrupted the air, and destroyed the health of the scholars. In the king's breve, the townspeople are ordered to repair and mend the floor or pavement of the town, in every street and lane; to remove all filth lying in them, also the hogs which did increase it; and the sheriff was to cause the burghers to do the like before their doors. Six years afterwards, another complaint was made to the king, that the regrators burned stinking fat and suet before their doors, which so corrupted the air, that the scholars were often sick, and by the smell brought into infirmities. Edward III compelled ecclesiastical persons to repair and clean before their doors, as well as laics, from which they had claimed an exemption." "In 1338 a complaint was again made to the king that dung, garbage, and other filthiness lying in streets, lanes, and alleys, had so much infected the air, that not only nobles, but others of inferior note did decline coming near the town, and scholars and burghers were overtaken with infirmities of body, and many had died." Any one who will examine the ancient statutes of the streets of the city of London, will find that in the middle ages passengers were exposed to the same annoyances as those which even in modern times disgraced the capital of the north. The minuteness with which offences against cleanliness are particularised, is sufficient proof that these regulations were made to remedy existing evils.

Although, as we have seen, the people of England for ages shewed but little antipathy to the existence of smoke in their dwellings, they were curious in their choice of the particular fume. Coal smoke was especially abhorred. Coal first appears as an article of commerce about the end of the twelfth century; and about a hundred years later, it was imported into London for the use of brewers, dyers, smiths, and others. Up to this time wood, turf, peat, and charcoal were the only articles of fuel. The coal innovation produced a powerful outcry; it was discovered that its fumes corrupted the air, and were prejudicial to health. The citizens complained through parliament to the king in 1306, and petitioned for the prohibition of its use. The consequence was a royal proclamation, and afterwards a commission to ascertain who burnt sea-coal within the city and in the neighbourhood, the first offence being punishable by fine, and the second by the demolition of the furnaces. The custom, however, continued without any abatement in the opposition it had evoked, and at last it was made a capital crime. Documents still exist proving that a man, in the reign of Edward I, was actually tried, condemned, and executed for burning coal in the city of London. The opponents of the smoke nuisance were certainly in earnest in those days.

Sir William Petty, writing about the year 1660, attributes the increased unhealthiness of London to the universal use of sea-coal, which he says was little used sixty years before, "For I have heard," he writes, "that Newcastle is more unhealthful than other places, and that many people cannot at all endure the smoak of London, not only for its unpleasantness, but for the suffocation which it causes."

Other and more potent causes of disease than the smoke of the sea-coal existed without the walls of the ancient city. Although on the north side, as Fitzstephen writes, were "fields for pasture and open meadows very pleasant, into which the river waters do flow, and mills are turned about with a delightful noise", the greater part of the suburbs of London consisted of marshy ground. The ancient history of Rahere, the founder of St. Bartholomew's Hospital, informs us, when speaking of its site, "Truly thys place (aforn his clensynge) pretendid noone hope of goodnesse. Right uncleane it was; and as a maryce dunge and fenny, with water almost ev'y tyme habowndynge." Moorfields was a vast swamp across which the citizens built causeways, by which they reached the suburban villages of Hoxton and Iseldon, and where, in the winter time, "the young men sported on the ice, binding to their shoes bones and holding stakes in their hands, headed with sharp iron, which sometimes they strike against the ice, and going on with such speed, as doth a bird in the air, or darts shot from some warlike engine," as we read in the account left by the worthy monk of Canterbury whom we have so often quoted. It was not until the sixteenth century that "this fenne or moore" was converted into "maine and hard ground." On the west, Westminster was surrounded by marshy land; occasionally high tides swept over Parliament Street, and in consequence of such inundations knights and barons have riden on horseback into

Westminster Hall. From the abbey to Chelsea the country was again a swamp; as late as seventy years since, snipes were shot over the sites of Eccleston and Warwick Squares. Besides these, there were the great marshes of Essex bordering on the Thames and Lea. When all these sources of malaria are taken into consideration we need not wonder at the "hoat agues" and fluxes which we so frequently read of in the Chronicles. The father of English medicine has occupied a large part of his great work in treating of the intermittent fevers of London, which appeared as regularly as the temperature of the marshes in its neighbourhood was changed by the spring tide sun and the autumn breeze. From being one of the most common, ague, in the present day, has become comparatively a rare disease in the metropolis; and there is the best ground for believing that it, with all its kindred of periodic affections, might be completely banished the community.

Before quitting the subject of early English habitations, we must allude to a practice which seems to have been peculiar to the nation. It was that of strewing the floors of their rooms with sedge or fresh rushes, or, when these were not procurable, with straw and hay. This ancient custom, which obtained in Anglo-Norman times, as the account of the housekeeping of Thomas à Becket proves, continued to the Tudor era. In the frequently quoted letter of Erasmus to Dr. Francis, the physician of Cardinal Wolsey, the continual plague and sweating sicknesses are partly attributed to the filth and slovenliness engendered by this usage. "As to the floors," he says, "they are usually made of clay, covered with rushes that grow in the fens, which are so slightly removed now and then, that the lower part remains sometimes for twenty years together, and in it a collection of beer, stale food, and other filthinesses not to be named. Hence, upon a change of weather, a vapour is exhaled very pernicious to the human body." From the same letter we learn that, although large windows had been introduced, the sides of the rooms being glazed with small panes, ventilation was but little improved,—the air obtained entrance only through chinks, the main object being to admit light and exclude wind.

There was one circumstance connected with the huts of our forefathers which we are tempted to regard as their only advantage,—it was, that they were easily burnt down. Despotic as the measure seems, we cannot wonder at the curfew. Fires had become so frequent in London, in the times of the Plantagenets, that Richard I. ordered that, in future, all houses in the city should be built to a certain height of stone, and covered with slates and burnt tiles. Yet up to the period of the great conflagration of September, 1666, wood was the chief material employed by the lower and middle orders. That great event in sanitary history, which destroyed the dark lanes and noisome alleys, where plague and fever had lurked for ages, ushered in a new epoch. In the whole history of our country no temporary calamity has ever been attended with such lasting benefit. Yet, although the improvements in the new city were great, they were not what they might have been. Had not selfish councils and vested interests interfered, London would, at the command of England's greatest architectural genius, have risen from her ashes to rival in magnificence the capitals of oriental despotism, and to surpass in beauty the fairest cities of Italy.

We must not linger to give a detailed account of the food of our ancestors. We can only indicate two great errors in the diet of even the better classes, which must conjointly have been productive of disease. One was, the constant consumption, for a great part of the year, of salted provisions; the other, the sparing use made of fresh vegetables and fruit. Peas and beans were almost the only vegetables used for centuries after the Norman Conquest, besides those chiefly employed for their flavouring qualities, such as potherbs, onions, leeks, and garlic. In the thirteenth century, as may be gathered from the household roll of Bishop Swinfield,* a great part, both of the butcher's meat and of the venison consumed, were salted; and in the reign of Henry VII. fresh meat was a luxury untasted even by gentlemen attending upon a powerful noble, except between Midsummer and Michaelmas. In the times of the later Stuarts the Martinmas beef, as the winter stock of salt provision was called, was universally laid in by families in the beginning of November.† We learn from the authority above quoted that the major part of the fruit brought to the tables of the great was foreign,—as dried figs, almonds, raisins, and nuts. Greens were used pickled or salted. Not only the potato, but the turnip, are of comparatively modern introduction. In the letter of Erasmus before referred to, he alludes to the prejudicial effect of the immoderate use of salt provisions on the health of the English. We need only add, that deaths from scurvy were always returned in the earlier bills of mortality, and in the reign of Charles II. were considerably on the increase.

* A Roll of the Household Expenses of Richard de Swinfield, Bishop of Hereford. Edited by the Rev. J. Webb. Camden Society: 1855.

† Macaulay's History of England.

At the commencement of this article we asserted that eras of pestilence had been emphatically the eras of hygienic measures. One or two more illustrative facts will perhaps give it a fitting termination. In what were the suburbs of ancient London, there is a district which still preserves something of its former air of solitude and quiet, amid the noise and tumult which have for five centuries been gradually gathering round it. In the reign of the third Edward, it was a piece of waste land belonging to the brethren of St. Bartholomew's Hospital, and situated outside the bars of Smithfield, at some distance from the city wall. When the black plague, after advancing with rapid strides over Europe, numbering in every community its victims by tens of thousands, had at length reached the sunny western shores of this island, there were in London two public spirited men who, foreseeing the fate that overhung the doomed city, determined to provide the means of extramural sepulture. These were Sir Walter Manny, the noble Hainault knight, companion-in-arms of Edward and the Black Prince, and Ralph Stratford, Bishop of London. Two pieces of land, adjoining each other, were purchased; that by the bishop in 1348 was called "No Man's Land," and in Stow's time was a fair garden, retaining the name of Pardon Church-yard ; that by Sir Walter, in the following year, was named Spittle Croft, from its being the property of the hospital, and was in extent thirteen acres and a rod. Both these fields were duly consecrated for burial. In the latter, Stow adduces not only the charters of Edward III, but also an inscription on a stone-cross erected in the church-yard, to prove that fifty thousand persons, during that fearful epidemic, found their final resting-place. For twenty years the ground was used as a burying-place for poor people and travellers, according to the benevolent will of the purchaser, who built there a chapel, and afterwards founded a priory of Carthusian monks ; adding to their possessions Pardon churchyard, long afterwards the last home of those who had forfeited their lives to the law, or had desperately ended them. After the dissolution of monasteries, the house, "a very large and goodly mansion, beautified with spacious gardens, walkes, orchards, and other pleasures, enriched with divers dependencies", was purchased of the Earl of Suffolk by Thomas Sutton, for the purpose of founding a hospital for eighty poor persons and forty scholars. Such, in few words, is the history of the Charterhouse, a locality endeared to every lover of modern English literature by the truthful fiction of Thackeray and the classic page of Washington Irving.

We have here, then, as early as the fourteenth century, a recognition of the necessity of extramural burial, at least during a time of great mortality. The old historian, so often quoted, expressly says that Sir Walter Manny made his purchase "in respect of danger that might befall in this time of so great a plague and infection." It was not so much out of care for the dead as on account of harm that might accrue to the living, that he carried his purpose into execution.

It is a well-known fact that in succeeding epidemics those vast receptacles of the dead—the plague pits—were made at a distance from the habitations of the people, and even in country villages it was ordered, in Queen Elizabeth's time, that places should be appointed apart in each parish for the burial of such as should die by pestilence. The testimony of one of the early bishops of the Reformation has been lately adduced in a court of law in favour of the practice of extra-urban sepulture.

In connection with the subject of the black death we may add, that we would refer any reader, who may be sceptical as to the amount of the depopulation it produced, to an article by Mr. Amyot in the twentieth volume of *Archæologia*, on the population of English cities in the time of Edward III. It is worth noticing that, within a comparatively short period after its subsidence, the king granted its first charter to St. Bartholomew's Hospital. A similar coincidence occurs in the history of St. Thomas's; it was purchased by the citizens in the year succeeding the last epidemic of sweating sickness, 1552, for "poor, impotent, lame, and diseased people." For centuries these were the only two institutions for the reception of the sick of all diseases in London.

It was at the time of plague visitations that the excellent "statutes of the streets of the city against annoiances" were put in force. Thus, in 1656, the College of Physicians, by royal authority, recommended "that the statutes and good orders made and formerly published against common beggars, against *plaies,* bowling allies, inmates, tippling houses, lestalls, against the sale of corrupt flesh or fish, may be revived and strictly executed, and that the skavengers in generall, and euery particular householder take care for the due and orderly cleansing of the streets and priuate houses, which will auaile much in this case."

Then follows a direction for the extermination of "dogges, catts, conies, and tame pidgeons, and that no swine may be permitted to range up and down the streets as they frequently doe, or rather, not to keepe any at all;" the notion of the time being that infection was capable of being conveyed by domestic animals.

Again, in the orders for health, it is enacted that "every householder do cause the street to be daily pared before his doore, and so to keep it cleane swept all the week long. That the sweeping and filth of houses be dayly carried away by the rakers, and that the raker shall giue notice of his comming by blowing of a horne, as heretofore hath beene done. That the laystalls bee remoued as farre as may be out of the city, and common passages, and that no night man or other be suffered to empty a vault into any garden neere about the city." These are succeeded by orders against the sale of unwholesome meat, fish, and musty corn.

Similar directions were published at each visitation, together with others for providing necessaries and medical attendance for the sick, for burning such clothes and bedding as had been used by them, for which, in cases of poverty, allowance was ordered to be made, and against the sale of such articles as were believed to be capable of harbouring the *materies morbi.* As may be supposed, the grand error was the barbarous and useless shutting up of infected houses, by which every chance of escaping the disease was cut off from those who were immured, and, as the prodigious mortality shows, without the least good effect in lessening the spread of the epidemic. Yet this was done by the direction and under the sanction of the men of science of the day, and is the best proof of that lamentable ignorance of the necessity of free ventilation in combating disease to which we have before alluded. But the year 1660 gave birth to the Royal Society; the era of Newton, and Boyle, and Halley, had arrived; the philosophy of Bacon was at length put to the test; and an impulse was given to the advance of science, whose force the roll of years and the succession of generations have served but to augment. For the nineteenth century it has been reserved to apply her results to the requirements of social existence. Although in the city—so dear to all Englishmen—we no longer look for the quaint picturesque of a former age,—the pointed roof, the overhanging gable, and the grotesque carving,—yet, following the guidance of advancing knowledge, and tutored by the experience of the past, we do and shall hail within her a healthier, happier population than existed in the time when her maidens sported round the May-pole on Cornhill, and her pale students sauntered in the summer twilight beneath the green foliage shadowing the pleasant walks about Gray's Inn.

